# MicroRNA Expression and Regulation in Human Ovarian Carcinoma Cells by Luteinizing Hormone

**DOI:** 10.1371/journal.pone.0021730

**Published:** 2011-07-12

**Authors:** Juan Cui, Joanna B. Eldredge, Ying Xu, David Puett

**Affiliations:** 1 Department of Biochemistry and Molecular Biology, University of Georgia, Athens, Georgia, United States of America; 2 Institute of Bioinformatics, University of Georgia, Athens, Georgia, United States of America; 3 College of Computer Science and Technology, Jilin University, Changchun, Jilin, China; University of Pennsylvania School of Medicine, United States of America

## Abstract

**Background:**

MicroRNAs have been widely-studied with regard to their aberrant expression and high correlation with tumorigenesis and progression in various solid tumors. With the major goal of assessing gonadotropin (luteinizing hormone, LH) contributions to LH receptor (LHR)-positive ovarian cancer cells, we have conducted a genome-wide transcriptomic analysis on human epithelial ovarian cancer cells to identify the microRNA-associated cellular response to LH-mediated activation of LHR.

**Methods:**

Human ovarian cancer cells (SKOV3) were chosen as negative control (LHR−) and stably transfected to express functional LHR (LHR+), followed by incubation with LH (0–20 h). At different times of LH-mediated activation of LHR the cancer cells were analyzed by a high-density Ovarian Cancer Disease-Specific-Array (DSA, ALMAC™), which profiled ∼100,000 transcripts with ∼400 non-coding microRNAs.

**Findings:**

In total, 65 microRNAs were identified to exhibit differential expression in either LHR expressing SKOV3 cells or LH-treated cells, a few of which have been found in the genomic fragile regions that are associated with abnormal deletion or amplification in cancer, such as miR-21, miR-101-1, miR-210 and miR-301a. By incorporating the dramatic expression changes observed in mRNAs, strong microRNA/mRNA regulatory pairs were predicted through statistical analyses coupled with collective computational prediction. The role of each microRNA was then determined through a functional analysis based on the highly-confident microRNA/mRNA pairs.

**Conclusion:**

The overall impact on the transcriptome-level expression indicates that LH may regulate apoptosis and cell growth of LHR+ SKOV3 cells, particularly by reducing cancer cell proliferation, with some microRNAs involved in regulatory roles.

## Introduction

Ovarian cancer, the most lethal gynecological cancer and second only to endometrial cancer in the number of female reproductive track and organ cases diagnosed, led to approximately 21,880 new cases in the U.S. in 2010 with about 13,850 deaths [1]. The high morbidity is attributable in large part to a lack of specific symptoms, thus delaying prognosis until advanced stages of the disease. The 5-year survival rate of ovarian cancer in later stages is 16–28% according to the International Federation of Gynecology and Obstetrics [2]. Consequently, the majority of ovarian cancer research has been focused on discovering screening methods for early diagnosis and identifying the possible contributing factors for prevention of the disease.

A high correlation between ovarian cancer risk and altered reproductive cycles has been reported in many clinical studies. For example a higher risk is associated with peri- or post-menopause [3] and incidences of infertility [4], while a decreased risk correlates with pregnancy, oral contraceptive use, hysterectomy and tubal ligation [4]. In addition, the elevated level of the gonadotropins, luteinizing hormone (LH) and follicle-stimulating hormone (FSH), was consistently found in cysts and the peritoneal fluid of patients with ovarian cancer or borderline ovarian tumors, compared to benign cysts or benign tumors [5], which has attracted considerable attention, along with the status (positive or negative) of the LH receptor (LHR), a member of the G protein-coupled receptor family, in the cancer. There are suggestions that LH could potentially serve as a cause or contributory factor to the development or progression of ovarian cancer [6], [7], while other clinical reports show that there are no clear trends that using gonadotropins to treat infertility will increase the risk of ovarian cancer [8], [9]. With such controversy [10], [11], [12], [13], elucidation of the downstream processes of LHR is imperative to discerning the ramifications of increasing levels of LH in LHR+ cancer cells.

We have reported earlier [14] that the phenotypes of the LHR+ cells, but not the LHR- cells, exhibited reduced proliferation, migratory and invasive properties in response to LH, and have shown elsewhere the consequent changes in transcriptomic level expression [15]. The present study is focused on elucidating the effects of LH on microRNA expression and regulation. This class of small non-coding RNAs has recently gained considerable interest in their involvement in cancer development and progression via regulation of key cancer genes [16], [17]. Many microRNAs are found to be androgen related, and their deregulation has correlated highly with initiation, progression and prognosis of human cancers [18], [19], [20], [21], [22], [23], [24], [25]. Given the hypothesis that many of the myriad changes can be observed from gene expression profiles of LH-mediated LHR activation in a human ovarian cancer cell line (SKOV3), we applied a large-scale transcriptomic analysis on the cells using an ovarian disease-specific array. Our present analyses include three major steps: a) comparative analysis on microRNA and mRNA expression in response to LHR expression and activation in SKOV3 cells, b) computational prediction of microRNA/mRNA regulation pairs, in conjunction with experimental validated information and c) functional analysis on target mRNAs to infer the major role of their regulatory microRNA.

## Materials and Methods

### Choice of Cells and Microarray Analysis via Ovarian-Cancer-Specific Array

The human SKOV3 ovarian carcinoma cell line [14], which does not express LHR, was chosen as a negative control in this study. The cells were stably transfected to express about 12,000 LHRs per cell, and the hormone binding affinity (K_d_) of human chorionic gonadotropin (CG) was 0.3 nM [14]. Human LH and CG were obtained from Dr. A.F. Parlow (National Hormone and Peptide Program, Torrance, CA). Total RNA was extracted from the cells following the same protocol described elsewhere [14], [15] and then subjected to microarray analysis using the Almac Diagnostic Ovarian Cancer DSA™ research tool (Durham, NC). Detailed procedures can be found at http://www.almacgroup.com. The tailored ovarian-cancer array measures over 100,000 transcripts on one chip, including those solely expressed in ovary and large numbers of novel ones that are not included in other standard microarrays (e.g. tissue or disease-specific splice variants, inherent antisense transcripts, and non-coding RNA obtained by the in-house sequencing analysis at Almac). The comparison of the array content between DSA and others can be found at http://www.almacgroup.com/wp-content/uploads/Ovarian_Cancer_DSA_techsheet2.pdf. Expression profiling was done on the control SKOV3 cells, the LHR+ SKOV3 cells, and the LHR+ cells incubated with LH for 1, 4, 8, and 20 hours [14], respectively, with three replicates for each of these six variable groups. All data is MIAME compliant and the raw data has been deposited in the GEO database with accession number GSE27328.

### Data Processing and Statistical Analysis

Since the ovarian DSA is a high-density platform including only perfect match (PM) probes, the transcript expression was computed using the PM-only normalization algorithm designed by Almac Diagnostics (http://csbl.bmb.uga.edu/~juancui/Publications/OvCan2011/PMonlyQC-documentation.pdf), which is similar to MAS5 (Affymetrix). Transcripts with the maximum intensity among all 18 samples lower than 10 were removed due to the extremely low signal-to-noise ratio, and the geometric mean expression of each transcript was calculated from the triplicates in each group. Through statistical analyses with ANOVA and Mann-Whitney test, only genes with a differential expression change of more than 1.5-fold, with the P-value< = 0.05 adjusted for multiple test by R package, were accepted for further analysis.

### microRNA Target Prediction

MiRanda [26] and TargetScan [27] were used for predicting the microRNA targets from the complete human genome. In addition, the Spearman's rank *Rho* coefficient between expression levels of the microRNA and their targets was calculated based on the following. Given X_i_ and Y_i_ with *i* = 1..n representing the expression profiles of microRNA and mRNA, where n = 6 represents six distinct cellular states in order of LHR−, LHR+,LH1, LH4, LH8 and LH20. The expression level was then converted to ranks x_i,_ and y_i_, respectively. The difference, d_i_ = x_i_−y_i_, between the ranks of each observation was calculated, and the coefficient is given by 
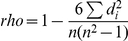
, where *rho>0*, *rho<0 and rho = 0* represent a positive, negative or no regulatory effect between microRNA and mRNA. Given the Null hypothesis H_0_ that x_i_ and y_i_ are independent, a two-tailed test was conducted where the p-value is given as 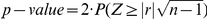. Those correlated pairs with modestly relaxed significance (|rho|>0.8, *p*-value< = 0.05) were the focus for further analysis.

## Results

### Altered microRNA and mRNA Expression Associated with LHR Expression and Activation

Out of 132 microRNAs that were selectively profiled on the array, 65 were identified to exhibit differential expression. Among them, 17 are differential in LHR+ SKOV3 cells vs LHR- SKOV3 cells, i.e. negative control, including six upregulated (miR-101-1, -101-2, -199b, -559, -573 and -7-3) and 11 downregulated (miR-103-2, -200c, -151, -29c, -301b, -548a2, -552, -561, -566, -613 and -642) (Table S1a). Following incubation with LH, 57 microRNAs were differentially expressed, including the most highly expressed microRNAs, miR-21, -200c, -593, -103-1 and -124-3 (Table S1b).

**Figure 1:**
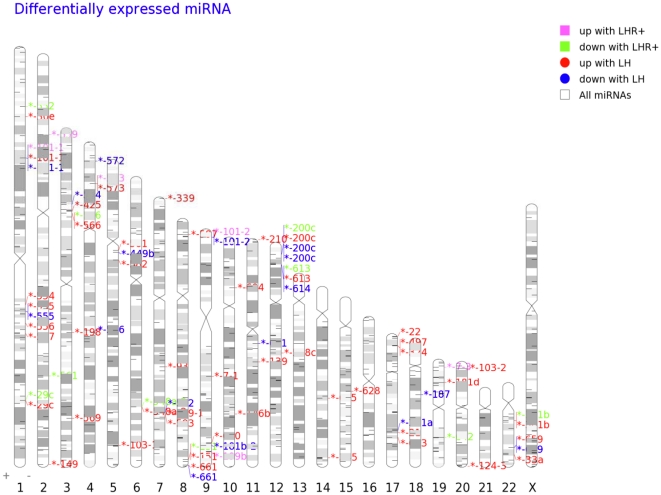
Mapping of differentially expressed microRNAs to the human genome.

The details of microRNA information were obtained from miRBase [28]. Of the 65 microRNAs, 19 are overlapping with intergenic regions, 45 are overlapping with introns and one with an exon, while 34 were found on the reverse strand (Table S2). Gene loci were then obtained from Ensemble (hg18). Figure 1 shows the mapping of microRNAs to the human genome. According to the microRNA clusters annotation in miRBase, i.e. neighboring microRNAs within the pre-defined distance of 10 kb, seven clusters were identified due to their inclusion of at least one differentially expressed microRNA, namely miR-93, -301b, -200c, -425, -29, -449b and -30e. Table S2 shows the impacted regions with the differentially expressed microRNAs within a less restricted distance range. Some of the differentially expressed microRNAs have been aligned to fragile sites of the human genome where the genomic variants were evidently associated with certain types of cancer [29], [30]. For example, the loss of 11p15 (covering miR-210) is found in ovarian cancer [31] and 17q23 (covering miR-301a and miR-21) is amplified in breast cancer [32], as well as those reported in [33], [34]. It should be noted that the SKOV3 cancer cells may have already undergone some copying abnormalities, such as the amplification of miR-21 and the loss of miR-210, which may contribute to the observed gene expression level in the control cancer cells. Although this cannot be determined from the present data, our question here, instead, is whether or not LH induces further transcriptional regulation on any of these microRNAs.

**Figure 2:**
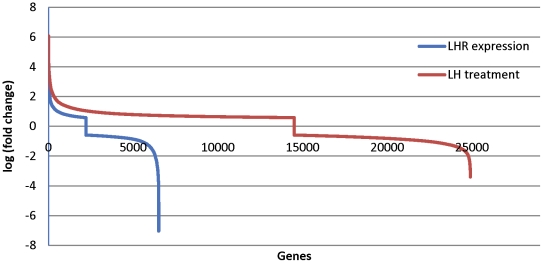
Distribution of the expression change of mRNAs in this study as cells express LHR and are activated by LH.

Through the same differential analysis, we have identified 2,210 up-regulated and 4,297 down-regulated mRNAs in LHR+ SKOV3 cells, respectively. The expression change distribution is given in Figure 2. These genes are intimately involved in cell division, DNA replication and transcription, while the genes involved with carbohydrate transport/metabolism and lipid metabolism are only reflected by down-regulated genes. During the 20 h exposure to LH, a total of 14,903 mRNAs exhibited elevated expression at one of the time points, which extend the above functions to posttranslational modification, RNA processing and modification, intracellular trafficking and secretion, signal transduction mechanisms and coenzyme metabolism, while 10,389 mRNAs were down-regulated, reflecting the cellular defense mechanism. The enriched pathway analysis shows that LHR expression in SKOV3 cells may have a positive impact on cellular gap junctions and relevant growth signaling pathways, while moderately suppressing apoptosis, mismatch repair and the Ras-Independent pathway in NK cell-mediated cytotoxicity, which is overall an advantage to cell growth. LH, subsequently, regulated gene expression involved in the cell cycle, p53 and VEGF signaling, gap junction, immune responses and the complement and coagulation cascades, as well as on a few metabolic pathways. The transcriptome expression analysis reflects those pathway alterations that support the phenotypes observed in our previous study [14], as well as many others [15].

### Prediction of microRNA/mRNA Regulation Pairs

**Figure 3:**
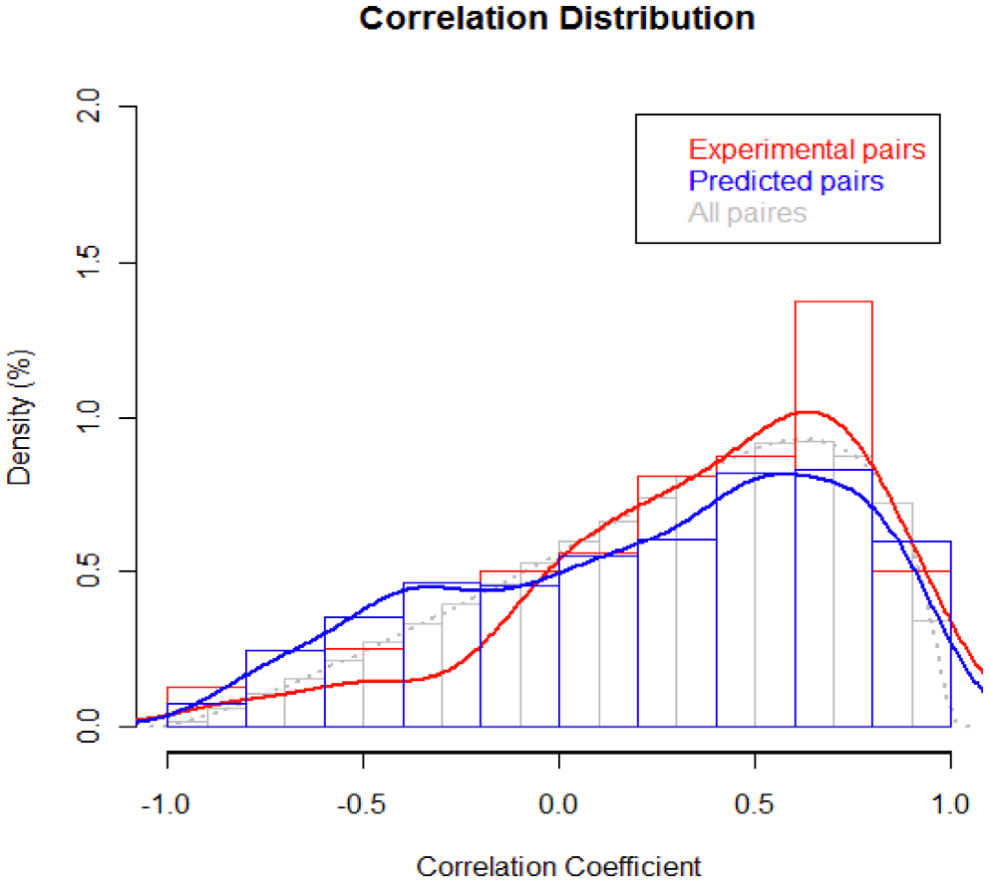
Distribution of the correlation coefficients between microRNA and mRNA targets. (The blue plot represents the predicted microRNA/mRNA pairs and the red plot represents the experimental validated pairs; the background plot in gray represents all pairs used for analysis in this study).

Inference of microRNA function can be approached by investigating the proteins and RNAs associated with the biological processes in which their mRNA targets are involved. The following strategy was adopted to first predict the potential mRNA target(s) for each microRNA. Spearman correlation analysis was performed between the expression of 65 differentially expressed microRNAs associated with LHR expression and activation and the expression of 60,860 well-annotated mRNAs across all sample groups (Materials and Methods). Among all possible microRNA-mRNA pairs, significant correlations were detected using (|*rho*|>0.8, *P*-value<0.05), which includes 62,150 and 931,009 pairs of negative and positive correlations, respectively. Figure 3 shows the distribution of the rho coefficient of both experimental and predicted microRNA/mRNA pairs against all calculated pairs, which indicates the significance of the interactions.

**Figure 4:**
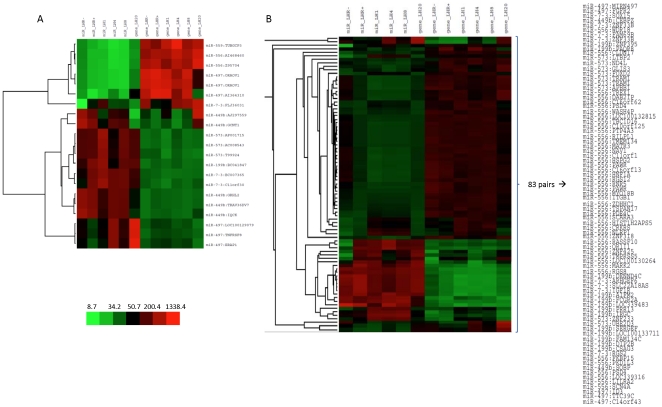
The heatmap of the most correlated expressions between microRNA and mRNAs. **A**: pairs with the highest negative correlation in expression, and **B**: pairs with the highest positive correlation in expression (|rho|>0.99).

**Figure 5:**
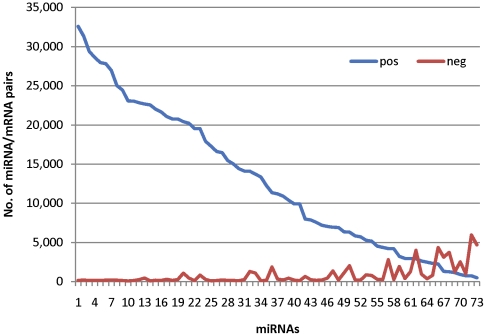
Comparison between the positively and negatively correlated pairs for each differentially-expressed microRNA (73 probes are involved for 65 unique microRNAs).

We then examined those pairs of particular interest, with the mRNAs differentially expressed in the LHR+ SKOV3 cells, i.e. LHR+ and the same with LH incubation for 1, 4, 8 and20 h, since our focus is on those that respond to LHR expression and activation. In total, 19,105 negatively and 211,893 positively correlated pairs are included, involving 19,154 unique mRNAs. Figures 4A and 4B illustrate the top correlated pairs from each group, most of which have not yet been experimentally validated. Interestingly, more positively correlated microRNA-mRNA pairs than negatively correlated pairs were observed for all microRNAs with the exception of nine, miR-181B2, miR-582, miR-497, miR-559, miR-561, miR-101-1, miR-187, miR-572 and miR-301A (Figure 5). The prevalence of high correlations may indicate that the microRNA is intronic or upstream to the correlated gene since they may share the same regulatory element and be transcribed together, and thus do tend to predict physically proximate pairs. The highly negative correlations are expected to indicate possible biochemically important interactions.

We further examined the expressions between microRNA and their host genes, which are generally concordant [35]. Among the 65 differentially expressed microRNAs, 13 intronic ones were observed that are highly or moderately correlated with their host genes, such as mir-555/ASH1L, mir-22/C17orf93, mir-198/FSTL1, mir-561/GULP1 and mir-564/KIF15, 18 poorly correlated and four negatively correlated with their host genes (Table S3). One possible explanation for the poor correlations is that the microRNAs may have their own regulatory elements rather than sharing the same with their host genes. To confirm this possibility, one can search for the transcriptional features in flanking sequences of microRNA precursors such as transcription start sites (TSS), CpG islands and transcription factor binding sites (TFBS), compared to the same analysis on the host genes. The very few observed negative correlations may be attributable to the interference by the diverse expression of splice variants of the same gene measured in this study. Of course, one cannot exclude the possibility that some microRNAs may regulate their host gene through the mRNA degradation machinery.

To ensure that the microRNA-correlated genes are direct targets instead of downstream genes, two popular programs, MiRanda [26] and TargetScan [27], were applied to predict miRNA targets. 584 genes were predicted as potential targets of the 65 microRNAs (see detailed list in Table S4). Although not all predicted pairs possess high correlations, it does show a trend that the percentage of predicted pairs decreased as the coefficients increase along its distribution. With the cutoffs used above, only 155 genes were retained as high-confident ones, i.e. negatively correlated with microRNA and differentially expressed, for functional analysis.

### MicroRNA Regulation Involved in LH Downstream Signaling in LHR+ SKOV3 Cells

For each microRNA, the function and pathway enrichment analysis was performed on its gene targets in order to infer the main function of the miRNA. From Table S1, it is indicated, for example, that miR-199b-5p participates in angiogenesis, nucleotide excision repair, the PDGF signaling pathway, the cadherin/Wnt/integrin signaling pathway, apoptosis and the MAPK signaling pathway, according to its regulation of the transcription/translation regulator and signaling transducer proteins enriched in its target gene set. MiR-101 is also involved in the Wnt/MAPK/cadherin signaling pathway, as well as hypertrophic cardiomyopathy (HCM), melanogenesis, the metabotropic glutamate receptor group III pathway and ubiquitin-mediated proteolysis. In addition, it may also regulate to some extent the mRNA for cyclic AMP (cAMP)-specific phosphodiesterase 4D (PDE4D), which was found to be highly up-regulated by LH in LHR+ cells [15]. MiR-29c is shown to mainly regulate ECM-receptor interaction, focal adhesion, collagen α chains and the integrin signaling pathway. The main regulation of miR-129 is that of angiogenesis, the Wnt signaling pathway, transcription regulation and cell junction. It is noteworthy that several of the microRNAs have the potential to regulate various tyrosine and serine/threonine kinases.

To further elucidate the microRNA regulation involved in LH-treated cancer cells, 70 experimentally validated targets of the 65 differentially expressed microRNAs were examined according to the miRecords database [36] (Table S1). Some of these genes were differentially expressed in the response to LH binding to LHR+ cancer cells, including those involved in the regulation of cell migration and proliferation (such as IRS1, IRS2, IL6R, TPM1, GLI1, BMPR2 and GRN), cell surface receptor-linked signal transduction (SOCS5 and RAF1), anti-apoptosis (FAS, MCL1 and SGK3) and transcription regulation (DNMT3B, GLI1 and EZH2), as shown in Table 1. Six genes exhibited highly correlated expression to their microRNAs (|*rho*|> = 0.7), including IRS1, IRS2 and RAF1 to miR-7-1, SGK3 and MTAP to miR-21 and GRN to miR-659. The negatively correlated microRNA/mRNA pairs are of more interest in reflecting a direct interaction. It is indicated from the expression changes that LH may impose a moderate positive regulation of cell proliferation, nucleotide metabolic process and cell surface receptor-linked signal transduction, and a negative regulation of apoptosis on ovarian cancer cells, in terms of the gene regulation through miR-7-1, miR-21 and miR-659. We also examined 54 oncogenes and tumor suppressor genes derived from literature sources [30], [37] (Table S5). As listed in Table 2, only six genes have experimental or predicted information. All the predicted targets are up-regulated except one: up-regulation of miR-21 and down-regulation of its target TPM1, a tumor suppressor gene. This may suggest a role of miR-21 in inhibiting apoptosis and consequently inferring a positive impact of LH on cancer development. Up-regulation of NF1, RB1 and SUFU shows a negative effect on cancer growth, consistent with our previous report [14].

**Table 1:** Differentially expressed microRNAs and their experimentally validated targets that also show significant expression change either with LHR expression or with LH treatment (*rho*: correlation coefficient between the expressions of microRNA and their targets, across all six conditions (LHR−, LHR+, LH1, LH4, LH8, and LH20)).

microRNAs	Fold change		Targets	Target Description	Fold change		*rho*	Function
	LHR+	LH+			LHR+	LH+		
microRNA 7-1	−1.3	1.8	IRS1	insulin receptor substrate 1	1.2	1.9	0.73	regulation of cell proliferation
			IRS2	insulin receptor substrate 2	1.3	2.0	0.72	
			RAF1	v-raf-1 murine leukemia viral oncogene homolog 1	−1.4	1.6	0.95	cell surface receptor linked signal transduction; apoptosis regulation
microRNA 101-1	1.6	−1.5	EZH2	enhancer of zeste homolog 2 (Drosophila)	1.8	−1.2	0.50	regulation of transcription; negative regulation of cell differentiation
			MCL1	myeloid cell leukemia sequence 1 (BCL2-related)	−1.1	1.7	0.01	anti-apoptosis
microRNA 21	−1.2	1.9	IL6R	interleukin 6 receptor	1.7	−1.4	0.14	regulation of apoptosis
			SGK3	serum/glucocorticoid regulated kinase family, member 3	−1.2	1.5	0.91	
			FAS	Fas (TNF receptor superfamily, member 6)	−1.3	1.5	0.36	
			MTAP	methylthioadenosine phosphorylase	−1.0	1.5	0.94	nucleotide metabolic process
			RP2	retinitis pigmentosa 2 (X-linked recessive)	−1.2	−1.5	0.17	
			BMPR2	bone morphogenetic protein receptor, type II (serine/threonine kinase)	1.2	1.6	0.44	type II serine/threonine-protein kinase receptor
			SERPINB5	serpin peptidase inhibitor, clade B (ovalbumin), member 5	−1.3	1.5	−0.12	cell motion
			TPM1	tropomyosin 1 (alpha)	1.6	−1.6	0.25	
			SOCS5	suppressor of cytokine signaling 5	−1.5	1.4	0.49	negative regulation of immune system; cell surface receptor linked signal transduction
microRNA 199b	2.0	1.3	LAMC2	laminin, gamma 2	−1.9	1.6	−0.48	cell division and chromosome partitioning; signal transduction mechanisms
microRNA 659	−1.1	1.5	GRN	granulin	−1.3	1.6	0.85	positive regulation of epithelial cell proliferation
microRNA 324	−1.2	1.5	GLI1	glioma-associated oncogene homolog 1 (zinc finger protein)	−2.3	−2.5	0.07	transcription regulation
microRNA 29c	−1.5	1.6	DNMT3B	DNA(cytosine-5-)-methyltransferase 3 beta	−1.1	−1.7	0.65	DNA metabolic process and modification
microRNA 29c	−1.5	1.6	FBN1	fibrillin 1	1.1	1.9	−0.02	extracellular matrix structural constituent
microRNA 103-1	−1.4	1.9	SERBP1	SERPINE1 mRNA binding protein 1	−1.2	−1.5	−0.12	regulation of anti-apoptosis and RNA metabolic process

**Table 2:** Correlation of expression between ovarian cancer-associated microRNA and their target oncogenes (↑ and ↓ represent, respectively, the up-regulation and down-regulation of gene expression in response to LH treatment, “−” means that no significant expression change was observed).

microRNA	Oncogenes	Gene name	Target (*rho*)	
Mir-21 (↑)	TPM1 (↓)	tropomyosin 1 (alpha)	known	−0.24
microRNA 103-1(↑)	NF1(↑)	neurofibromin 1	predicted	0.94
microRNA 7-3 (−)	RB1(↑)	retinoblastoma 1	predicted	0.68
microRNA 103-1(↑)	SUFU(↑)	suppressor of fused homolog (Drosophila)	predicted	0.89
microRNA 103-1(↑)	CDK6(−)	cyclin-dependent kinase 6	predicted	0.04
microRNA 181b-2(↓)	SOX5(−)	SRY (sex determining region Y)-box 5	predicted	−0.43

## Discussion

Numerous recent studies have reported that the global expression of microRNAs is deregulated in most cancers, including epithelial ovarian cancer [38]. We present here the first study demonstrating that LH regulates microRNA expression in LHR+ SKOV3 cells. With the continuous exposure of LH to the LHR+ cells, the highly correlated expression patterns observed between differential microRNAs and their target genes affirm the underlying predicted interactions and were then applied for inferring the involvement of microRNA regulation. One advantage of using a disease-specific array is the gathering of highly extensive mRNA data and microRNA information in ovarian cancer on the same chip which largely avoids the technical noise associated with profiling their expression in separate chips.

Prediction of microRNA and mRNA regulation pairs is clearly a crucial component in this study, which is mainly through correlation analysis in conjunction with collective computational prediction. We first note the limitations of correlation analysis in two regards: i) as the microRNAs fluctuate in the network, the relative expression change in their target mRNAs may not be concordant because of many other transcriptional regulation mechanisms, and ii) an excess of high positive and negative correlation pairs were observed, but most of them do not contain sufficiently complementary sequences to predict a target relationship, nor do they lie in physical proximity to each other. Clearly, correlations alone are not adequate for accurate prediction, but can be used as an auxiliary validation. Numerous computational algorithms have been recently developed for predicting microRNA/mRNA interactions based on information embodied in the sequence and structure [39], including those used in this study. It is known that microRNA binding to its target mRNA is through an Argonaute (Ago)-containing effector complex, referred to as RISC (RNA induced silencing complex), where Ago proteins plays a central role in recognizing and binding to target mRNAs [40]. It is therefore anticipated that a method taking into consideration the sequence or structure information of AGO proteins may significantly improve the prediction performance.

The results reported herein on the down-regulation of miR-200c concomitant with LHR expression and activation are particularly interesting in terms of a recent report on the analysis of microRNAs in tumor tissues from patients with stage I epithelial ovarian cancer [41]. It was found that down-regulation of microRNA-200c correlated with overall survival, but the patients may be more susceptible to relapse. It is well recognized that the microRNA family (200a, 200b, 200c, 141, 429) is important in regulating metastasis [42]. Another study found that miR-22 correlated with inhibition of cancer cell migration and invasion [43], so the down-regulated expression reflects the consistency with our observations that the addition of LH to LHR+ SKOV3 cells inhibited cell proliferation, migration, and invasion [14]. Upregulation of miR-21 was observed in our study, which may be caused by DNA hypomethylation as reported in vivo [44] and shows consistency with the ovarian steroids regulation (ICI-182780 and MPA treatment) in myometrial and leiomyoma cells [45]. In additional to those above, the most prominent and “hypoxia-responsive” miRNA, miR-210, was found to be unregulated by LH, which modulates the expression of genes promoting cell survival and tumor growth under hypoxic condition [46].

We have previously shown that ligand-mediated activation of LHR in the LHR+ SKOV3 cells activates second messenger responses, cAMP and inositol phosphates [14], which initiate protein kinase cascades that are involved with acute cellular effects, e.g. steroidogenesis in steroidogenic cells and mitogenic signaling [47], [48]. It was also demonstrated that LH reduces cell proliferation of LHR+ (but not LHR−) SKOV3 cells, as well as cell migration and invasiveness [14]. In order to elucidate the mechanism of this hormonal regulation, previous work has established that LH is responsible for the transcriptional regulation of a relatively large number of genes and pathways that could explain the underlying cellular responses [15]. In this work, microRNAs, as important gene regulators, were studied by profiling the expression patterns and analysis of their target genes; some of the microRNAs exhibit characteristics suggesting an important role in regulating downstream LHR signaling in ovarian cancer cells. Subsequent studies will be necessary to determine the specific function of each such microRNA.

In summary, although the role of LH in the progression of ovarian cancer is still controversial, it is well documented that LH-mediated signaling affects many microRNAs, genes and pathways in the LHR+ SKOV3 cells, and the indicative clues of inhibiting apoptosis and regulating cell proliferation may direct further research and help elucidate causes, detection and treatment of ovarian cancer.

## Supporting Information

Table S1 MicroRNAs differentially expressed in SKOV3 cells with LHR expression and activation (including expression fold change and target information).
(DOCX)

Table S2 14 pairs of closest neighbors from the differentially expressed microRNAs identified in our data.
(XLSX)

Table S3 Correlated groups of microRNAs and their host genes.
(XLSX)

Table S4 Predicted gene targets for each microRNA by both MiRanda and TargetScan.
(XLSX)

Table S5 Correlation between microRNAs and cancer genes.
(XLSX)
