# Supplementary material for: MicroRNA Expression and Regulation in Human Ovarian Carcinoma Cells by Luteinizing Hormone
Source: PLoS One. 2011 Jul 12;6(7):e21730. doi: 10.1371/journal.pone.0021730 (PMC3134471; doi:10.1371/journal.pone.0021730)
Supplement: Table S1 — MicroRNAs differentially expressed in SKOV3 cells with LHR expression and activation (including expression fold change and target information). (DOCX) [file pone.0021730.s001.docx]

Table S1. MicroRNAs differentially expressed in SKOV3 cells with LHR expression and activation (including expression fold change and target information)

A. Those affected by LHR expression.

| **LHR expression** | | **LH treatment** | | **microRNA** | **Known Targets** | **No. of Predicted Targets** | **Functionally relevant process** |
| --- | --- | --- | --- | --- | --- | --- | --- |
| Regulation | (FC) | Regulation | (FC) |  |  |  |  |
| up | 1.5 | up | 1.5 | microRNA 573 |  |  |  |
|  | 2.0 | None | - | microRNA 199b | LAMC2 | 39 | angiogenesis, nucleotide excision repair, PDGF signaling pathway, cadherin/Wnt/integrin signaling pathway, apoptosis and MAPK signaling pathway |
|  | 1.5 |  | - | microRNA 559 |  |  |  |
|  | 2.1 |  | - | microRNA 7-3 |  | 39 |  |
|  | 1.6 | down | -1.5 | microRNA 101-1 | MYCN, MCL1, ICOS, PTGS2, EZH2 | 84 |  |
|  | 2.0 |  | -1.8 | microRNA 101-2 |  |  |  |
| down | -1.5 | up | 1.5 | microRNA 151 |  |  |  |
|  | -1.2 |  | 2 | microRNA 103-2 |  |  |  |
|  | -1.5 |  | 1.5 | microRNA 29c | LAMC1, DNMT3A, DNMT3B, COL3A1, COL4A1, COL15A1, TDG, FUSIP1, COL1A1, COL1A2, COL4A2, FBN1, PIK3R1, CDC42 | 113 | ECM-receptor interaction, focal adhesion, integrin signaling pathway |
|  | -1.6 |  | 2.1 | microRNA 548a-2 |  |  |  |
|  | -1.6 |  | 1.5 | microRNA 566 |  |  |  |
|  | -2.3 |  | 1.5 | microRNA 613 |  |  |  |
|  | -1.5 | None | - | microRNA 301b |  |  |  |
|  | -1.6 |  | - | microRNA 552 |  |  |  |
|  | -1.6 |  |  | microRNA 642 |  |  |  |
|  | -2.2 |  | - | microRNA 561 |  |  |  |
|  | -1.9 | down | -1.6 | microRNA 200c |  |  |  |

B. Those affected by LH-mediated activation of LHR

| **LH treatment** | | **microRNA** | **Known Targets** | **No. of Predicted Targets** | **Functionally relevant process** |
| --- | --- | --- | --- | --- | --- |
| **Regulation** | **FC** |  |  |  |  |
| up | 1.9 | microRNA 103-1 | FBXW11, ICOS, SERBP1 | 61 | angiogenesis, hedgehog signaling pathway, parkinson disease, Wnt signaling pathway, MAPK signaling pathway, neurotrophin signaling pathway, cadherin signaling pathway, muscarinic acetylcholine receptor 2 and 4 signaling pathway, FGF signaling pathway |
|  | 2 | microRNA 103-2 |  |  |  |
|  | 1.5 | microRNA 124-3 |  |  |  |
|  | 1.5 | microRNA 129-1 | NOTCH1 | 52 | Angiogenesis, Wnt signaling pathway, transcription regulation, cell junction |
|  | 1.5 | microRNA 139 |  |  |  |
|  | 1.5 | microRNA 146b | MMP16 | 13 |  |
|  |  |  |  |  |  |
|  | 1.7 | microRNA 149 |  |  |  |
|  | 1.5 | microRNA 181d |  |  |  |
|  | 1.6 | microRNA 198 |  |  |  |
|  | 1.9 | microRNA 21 | TPM1, CDK6, TIMP3, PDCD4, SERPINB5, NFIB, PDCD4, CDKN1A, FAS, FAM3C, HIPK3, PRRG4, ACTA2, BTG2, BMPR2, SESN1, IL6R, SOCS5, GLCCI1, APAF1, SLC16A10, SGK3, RP2, CFL2, RECK, MTAP, SOX5 | 34 |  |
|  | 1.8 | microRNA 210 | EFNA3 | EFNA3,FAM116A |  |
|  | 1.7 | microRNA 22 | ESR1, PPARA,BMP7 |  |  |
|  | 1.5 | microRNA 30e | UBE2I | 188 genes |  |
|  | 1.5 | microRNA 324 | GLI1, SMO | 13 genes |  |
|  | 1.6 | microRNA 339 |  |  |  |
|  | 1.5 | microRNA 33a |  |  |  |
|  | 1.5 | microRNA 345 |  |  |  |
|  | 1.5 | microRNA 425 |  |  |  |
|  | 1.6 | microRNA 497 |  |  |  |
|  | 1.5 | microRNA 548c |  |  |  |
|  | 1.8 | microRNA 554 |  |  |  |
|  | 1.5 | microRNA 556 |  |  |  |
|  | 1.5 | microRNA 557 |  |  |  |
|  | 1.9 | microRNA 569 |  |  |  |
|  | 1.5 | microRNA 581 |  |  |  |
|  | 2.3 | microRNA 582 |  |  |  |
|  | 1.5 | microRNA 593 |  |  |  |
|  | 1.5 | microRNA 597 |  |  |  |
|  | 1.5 | microRNA 600 |  |  |  |
|  | 1.5 | microRNA 604 |  |  |  |
|  | 1.5 | microRNA 625 |  |  |  |
|  | 1.6 | microRNA 628 |  |  |  |
|  | 1.6 | microRNA 633 |  |  |  |
|  | 1.8 | microRNA 7-1 | IRS1, IRS2, EGFR, PAK1, RAF1 | 39 genes |  |
|  | 1.6 | microRNA 93 | E2F1, VEGFA, CDKN1A | 69 genes |  |
| down | -1.5 | microRNA 138-2 |  |  |  |
|  | -1.6 | microRNA 181b-2 | TCL1A, VSNL1, GRIA2, Aicda | 159 genes |  |
|  | -1.5 | microRNA 187 |  |  |  |
|  | -1.6 | microRNA 301a |  |  |  |
|  | -1.5 | microRNA 449b |  |  |  |
|  | -1.5 | microRNA 564 |  |  |  |
|  | -1.5 | microRNA 572 |  |  |  |
|  | -1.5 | microRNA 576 |  |  |  |
|  | -1.5 | microRNA 592 |  |  |  |
|  | -1.5 | microRNA 611 |  |  |  |
|  | -1.8 | microRNA 614 |  |  |  |
|  | -1.5 | microRNA 659 | GRN |  |  |
